# The Role of Family Environment in Depressive Symptoms among University Students: A Large Sample Survey in China

**DOI:** 10.1371/journal.pone.0143612

**Published:** 2015-12-02

**Authors:** Yunmiao Yu, Xiuxian Yang, Yanjie Yang, Lu Chen, Xiaohui Qiu, Zhengxue Qiao, Jiawei Zhou, Hui Pan, Bo Ban, Xiongzhao Zhu, Jincai He, Yongqing Ding, Bing Bai

**Affiliations:** 1 Department of Psychology, Public Health Institute, Harbin Medical University, Harbin, China; 2 Department of Endocrinology, Peking Union Medical College Hospital, Beijing, China; 3 Department of Endocrinology, Jining Medical College, Jining, China; 4 Medical Psychological Institute, SecondXiangyaHospital, CentralSouthUniversity, Changsha, China; 5 The First Affiliated Hospital, WenzhouMedicalUniversity, Wenzhou, China; 6 The fifth affiliated hospital, HarbinMedicalUniversity, Daqing, China; 7 The First Affiliated Hospital, HarbinMedicalUniversity, Harbin, China; Istituto Superiore di Sanità, ITALY

## Abstract

**Objective:**

To explore the relationship between family environment and depressive symptoms and to evaluate the influence of hard and soft family environmental factors on depression levels in a large sample of university students in China.

**Methods:**

A multi-stage stratified sampling procedure was used to select 6,000 participants. The response rate was 88.8%, with 5,329 students completing the Beck Depression Inventory (BDI) and the Family Environment Scale Chinese Version (FES-CV), which was adapted for the Chinese population. Differences between the groups were tested for significance by the Student’s t-test; ANOVA was used to test continuous variables. The relationship between soft family environmental factors and BDI were tested by Pearson correlation analysis. Hierarchical linear regression analysis was conducted to model the effects of hard environmental factors and soft environmental factors on depression in university students.

**Results:**

A total of 11.8% of students scored above the threshold of moderate depression(BDI≧14). Hard family environmental factors such as parent relationship, family economic status, level of parental literacy and non-intact family structure were associated with depressive symptoms. The soft family environmental factors—conflict and control—were positively associated with depression, while cohesion was negatively related to depressive symptom after controlling for other important associates of depression. Hierarchical regression analysis indicated that the soft family environment correlates more strongly with depression than the hard family environment.

**Conclusions:**

Soft family environmental factors—especially cohesion, conflict and control—appeared to play an important role in the occurrence of depressive symptoms. These findings underline the significance of the family environment as a source of risk factors for depression among university students in China and suggest that family-based interventions and improvement are very important to reduce depression among university students.

## Introduction

Depression is estimated to affect 3%–16.9% of individuals worldwide [1]. University students have a higher risk of developing depression than the general population [2,3]. In a recent study, depression was present in nearly one-third (a weighted mean prevalence of 30.6%) of all evaluated students, which constitutes a depression rate that is 9% higher than that observed in the general population [4]. Depression can influence the quality of life of university students, affecting their social and family relationships, academic productivity and physical functioning [5,6]. This decreases their ability and motivation to learn, resulting in poor academic performance and even dropping out of university [7]. Depression has consistently been identified as a significant risk factor for suicide attempts in university students [8,9]; therefore it is imperative to explore the factors influencing the development of depression in university students.

In China, about 75% of senior middle school students have the opportunity to obtain higher education. The rise in depression among college students (from about 5%–10% in 2002 to 24%–38% in 2011) has occurred as more young people pursue higher education [10]. University students are typically 18–23 years of age. Social scientists use the term “post-adolescence” to describe this period in university students [11]. Risk factors for adolescent depression include multiple and complex issues of personal characteristics and both family and school environments [12]. Among the potential risk factors of depression, family relations deserve particular attention since factors such as parental styles and family dynamics affect how the children develop [13].Studies have shown that family factors affect the development, maintenance and course of youth depression [14,15]. In general, depressed adolescents experience more negative family and parent–child relationships than normal adolescents.

Family environmental factors can be divided into hard and soft. Hard environmental factors include family structure, education level of the parents and the economic status of the family. An accumulating number of studies have described inconsistent results regarding the association of the hard family environment with depression in university students. First, undergraduates from single-parent families had lower scores of intimacy and emotional expression than those from complete families [16]. Second, university students born to mothers with a college education or higher have lower depression scores than those born to mothers with a lower level of education [9]. However, Parker et al. reported an association between depression and the highest level of maternal literacy attainment in male students [17], while Chang et al. failed to find an association between depression and the education level of the parents in university students [18]. Third, university students from families with a low economic status have a higher rate of depression than those from families with a moderate or good economic status [19–21]. However, one study did not find any association between the prevalence of depression and household income in medical students [22]. It may be that medical students tend to come from more affluent backgrounds and their future jobs are guaranteed. Hence, the relationship between the hard family environment and depression needs to be confirmed by investigating a large sample.

The soft family environment is divided into explicit factors (role of parent example, family regulation and rules, parent educational idea etc) and implicit factors (family culture, parent-child relationship, family member interaction etc). Soft environment is mainly social environment within the family, which has been considered an important factor regarding adolescent mental health [23]. Families operate as one of the major microsystems in creating and maintaining maladaptive behavior through multiple functional processes [24]. Among the soft family environmental factors, cohesion and conflict are the most important in predicting a healthy psychological adjustment for adolescents. Youth depression has been associated with high family conflict [25], low family cohesion [15] and marital discord, particularly marital conflict [14]. Sagrestano et al. reported that depression was related to the family environment in African American adolescents, even when controlling for source bias. Some reports have revealed that family cohesion is the most critical family variable in predicting depression in adolescents [26]. Eaton reported that high levels of negative family expression may have a negative impact on emotionality and emotional regulation [27].

Cultural background has also been associated with the occurrence of depression in adolescents [28].The Chinese traditionally emphasize familism and harmony and attach importance to blood ties and emotional connection. Chinese adolescents are subjectively willing to accept the influence of family but this is lost when university students leave the family home for the first time and reside with other students. This change may increase the risk of depression [29]. In addition, most Chinese university students are only children due to China’sone child policy and have to share a room with others for the first time when going to university. Consequently, university students are constantly faced with problems of crowded accommodation and have to adapt to a collective life. Additionally, due to the one child family structure, Chinese families are greatly concerned for their children. Parents tend to protect and spoil their children and are willing to tolerate mistakes, therefore Chinese university students tend to be more self-centered compared to former generations prior to the one-child policy. Upon attending university, however, they have to learn to get along with others. Furthermore, Chinese university students are faced with stern employment pressure and to lay a good foundation for future employment, they must strive to improve their academic achievement. University students have to manage challenges, such as fierce academic competition, strained interpersonal relationships and severe employment pressure, alone. The family is a key source of social support for Chinese university students in coping with stress. If students do not receive effective family support, then depression may increase. Thus we hypothesis that the family environment—especially cohesion and conflict—play a role in the psychological adjustment of Chinese university students.

This study aimed to clarify the relationship between hard family environmental factors and depression and to examine the relationship between soft family environmental factors and depression; and to evaluate the function of hard and soft family environmental factors in depression in a large sample of university students living in Harbin, China.

## Methods

### Sample size and sampling technique

To obtain a representative sample of university students in Harbin, we selected six universities at random. We weighted the distribution of samples from every university by the proportion of students who attended the university. We employed a stratified two-stage cluster selection of university students in full-time study. Then we stratified the sample into four grades (first, second, third and fourth year) and randomly selected classes from these grades. All students from the selected classes were invited to participate in the study. In total, we randomly selected 6,000 students from a total of 274,041 students in Harbin. Six thousand questionnaires were distributed and 5,479 were returned (91.3%). Excluding 239 invalid questionnaires (those with > 20% of the questions unanswered), 5,329 students successfully completed the screening questionnaire (88.8% completion rate). Postgraduate students were excluded, leaving a final number of 4,582 undergraduate students that were analysed statistically. The mean age of the participants was 20.79 years (standard deviation (SD) = 1.507).

### Procedure

Approval for this study was granted by the Ethics Committee of Harbin Medical University. All participants received a full explanation of the nature and purpose of the study. Confidentiality was assured and all related questions were answered. All participants were recruited directly in their respective classrooms. They were asked to take 40 minutes to fill the questionnaire. Participation was completely voluntary, with no motivation–economic or otherwise, and each participant provided written informed consent. The questionnaire was not distributed at the beginning or end of the semester, when students were under stress related to preparing for final exams and finishing projects.

### Instruments

The Beck Depression Inventory (BDI) is a self-report measure of depressive symptoms and includes 21 reliable and well-validated items [30]. Each item has a score range of 0–3, with a possible total score of 63. A score of 0–4 is considered normal, 5–13 borderline clinical depression, 14–20 moderate depression and 21–63 severe depression. The internal consistency (Cronbach α) for the BDI is high in many countries, ranging from 0.75–0.88[31] and was 0.851 in this study.

The Family Environment Scale Chinese Version (FES-CV), which was adapted for the Chinese population, was used to assess the soft family environmental factors [32]. The FES-CV contains 90 items, which are scored as true or false. There are ten subscales assessing different relational traits: (1) cohesion–the level of commitment, help and support among the family members; (2) expressiveness–the amount to which family members are encouraged to express their feelings; (3) conflict–the extent of open expression of anger and conflict between family members; (4) independence–the degree of esteem, self-confidence and independence among family members; (5) achievement orientation–the extent to which family members view general activities (such as study or work) as achievement-oriented or competitive; (6) intellectual-cultural orientation–the degree of interest in political, intellectual and cultural activities; (7) active-recreational orientation–the extent to which family members join recreational activities; (8) moral-religious orientation–the degree of emphasis on ethnicity, religion and value in family members; (9) organization–organization and structure for planning family activities and assigning responsibilities; (10) control–the extent to which family members use the rules and procedures to arrange their life. The FES-CV has good reliability and validity, except for three subscales: expressiveness, independence and moral-religious orientation [33]. These subscales were eliminated from our study, thus seven subscales in total were used. The Cronbach α of cohesion, conflict, intellectual-culture, organization, achievement, active-recreation and control subscale is 0.813, 0.807, 0.798, 0.764, 0.712, 0.726 and 0.708.

The self-designed questionnaire was used to evaluate hard family environmental factors and the socio-demographic variables. The hard family environmental variables included parental relationship, maternal literacy level, family economic status and family intact. The demographic variables included gender, age, grade, ethnicity, religion and the level of satisfaction with the chosen major.

### Data analysis

The statistical package for social science 18.0 (SPSS 18.0) program was used for statistical analysis. All tests were two-tailed and the significance level was set at p < 0.05. Differences between the groups were tested by the Student’s t-test and ANOVA was used to test continuous variables. The relationship between soft family environmental factors and BDI was examined by Pearson correlation analysis. Hierarchical linear regression analysis was conducted to model the effects of hard and soft environmental factors on depression in university students. In the regression model, gender, age, ethnicity, grade and satisfaction with chosen major were entered in the first block in order to control for potential confounding variables. In the second block, hard family environmental factors including family structure, family economic status, parent relationship and maternal literacy level were entered into the model. Finally, after controlling for sociodemographic variables and hard family environmental factors, the dimensions of the soft family environment were entered into the model.

## Results

### The distribution of depressive symptoms in university students

A total of 2,409 participants (52.6%) scored above the threshold for depressive symptoms; 1,871 (40.8%) scored in the range of borderline clinical depression, 384(8.4%) scored in the range of moderate depression and 154 (3.4%) scored in the range of severe depression. In total, 11.8% of students had symptoms that scored above the threshold of moderate depression.

### Sociodemographic data and BDI scores of different sociodemographic variables

Of the 4,582 participants, 2,299 were male and 2,283 were female; 4,873 were of Hans origin and 367 had another ethnic background. The numbers of participants in each grade were as follows: first grade, 1,369; second grade, 1,206; third grade, 1,290; and fourth grade, 717. The levels of satisfaction with chosen major were as follows: yes, 1,816; moderate, 2,440; no, 326. In total, 4187 students did not have any religious belief and 395 students had religious beliefs. No significant differences in BDI scores were found between gender, ethnicity or grades. The BDI scores were significantly affected by the level of satisfaction with chosen major and religion (Table 1).

**Table 1:** The participants’ sociodemographic data and BDI scores in different sociodemographic variables.

	Group	N (%)	BDI score	F/t	P
Gender				0.82	0.412
	Male	2299 (50.2)	6.26±6.13		
	Female	2283 (49.8)	6.41±5.94		
Grade				2.425	0.064
	The first	1369 (29.9)	6.38±5.86		
	The second	1206 (26.3)	6.52±6.39		
	The third	1290 (28.2)	6.42±6.16		
	The fourth	717 (15.6)	5.79±5.51		
Major satisfaction				86.208	<0.001
	Yes	1816 (39.6)	5.27±5.36		
	Moderate	2440 (53.3)	6.68±6.04		
	No	326 (7.1)	9.71±7.84		
Ethnicity				0.049	0.961
	Han	4258 (92.9)	6.33±6.06		
	Others	324 (7.1)	6.35±5.81		
Religion				4.745	<0.001
	No	4187 (91.4)	6.21±5.89		
	Yes	395 (8.6)	7.71±7.30		

### BDI scores according to different hard family environmental factors

We compared the BDI scores according to different hard family environmental factors (Table 2). The independent t-test showed a significant difference in depressive symptoms between family intact and non-intact groups (p < 0.05). Students in the non-intact family group scored significantly higher than students in the intact family group. ANOVA results showed significant differences between the poor family economic status group and the other groups (p < 0.05). Students with poor family economic status scored highest among the three groups. Parental relationship status significantly affected the BDI scores (p < 0.05); poor parental relationship groups scored higher than moderate and good relationship groups. The BDI scores were also significantly affected by the maternal and paternal literacy level. The higher levels of parental literacy were associated with lower levels of depressive symptoms(p < 0.05).

**Table 2:** BDI scores in different hard family environment.

Hard family environment		N (%)	BDI score	F/t	P
Family structure				2.108	0.035
	Intact	4188 (91.4)	6.28±5.97		
	Non-intact	394 (8.6)	6.95±6.77		
Maternal literacy				21.234	<0.001
	Primary school	857 (18.7)	7.56±6.89		
	Junior school	1275 (27.8)	6.48±5.97		
	Senior school	1597 (34.9)	6.11±5.86		
	College or higher	853 (18.6)	5.21±5.33		
Paternal literacy				15.566	<0.001
	Primary school	552 (12.1)	7.68±6.75		
	Junior school	1234 (26.9)	6.63±6.22		
	Senior school	1690 (36.9)	6.13±5.87		
	College or higher	1106 (24.1)	5.66±5.58		
Family economic status				28.374	<0.001
	Good	326 (7.1)	5.79±6.05		
	Moderate	3067 (66.9)	5.96±5.73		
	Poor	1189 (25.9)	7.46±6.56		
Parent relationship				53.994	<0.001
	Good	3667 (80.0)	5.88±5.66		
	Moderate	705 (15.4)	8.06±6.85		
	Poor	210 (4.6)	8.52±7.77		

### Relationship between soft family environmental factors (FES-CV) and depressive symptoms

We collected the FES-CV scores for each soft family environmental factor in university students (Table 3). Statistically significant linear relationships were found between each soft family environmental dimension and university students’ depressive scores (p<0.05). Among these variables, active-recreational orientation (r = –0.09, p<0.05), cohesion (r = –0.33, p<0.05), achievement orientation (r = –0.15, p<0.05) and organization (r = –0.19, p<0.05) were negatively related to the level of depression. Conversely, conflict (r = 0.29, p<0.05), intellectual-cultural orientation (r = 0.06, p<0.05) and control (r = 0.29, p<0.05) were positively associated with the depression level.

**Table 3:** Correlation between soft family environment (FES dimensions) and depressive symptoms.

	Cohesion	Conflict	Achievement	Intellectual- cultural	Active- recreational	Organization	Control
BDI	-0.331	0.288	-0.146	0.060	-0.087	-0.187	0.285

Note: Correlation is all significant at the 0.05 level (2-tailed); N = 4582.

### Hierarchical linear regression analysis of the relationship between family environment and depressive symptoms

The 17 family environment variables accounted for23.0% of thetotal variance of depressive symptoms (Table 4). The control variables accounted for 3.6%of the variance in predicting the levels of depression (F change _(6, 4571)_ = 34.10, p <0.01), and among them age (β = 0.06, t _(4571)_ = 2.78, p = 0.005), satisfaction with chosen major (β = 0.18, t _(4571)_ = 12.51, p<0.01), grade (β = -0.07, t _(4571)_ = -3.55, p< 0.01)significantly associated with depressive symptoms. With the entrance of hard family environmental variables the explaining variance increased to 6.8%.The variance in predicting levels of depression (F change _(4, 4567)_ = 31.18, p <0.01), family structure(β = -0.04, t _(4567)_ = -2.34, p = 0.019), maternal literacy level (β = -0.07, t _(4567)_ = -3.83, p <0.01), family economic status (β = 0.04, t _(4567)_ = 3.12, p = 0.002) and parent relationship (β = 0.11, t _(4567)_ = 7.42, p <0.01) were all significantly associated with depressive symptoms. Adding the soft family environmental factors contributed to an additional 16.2% of the variance in predicting depressive symptoms (F change _(7, 4560)_ = 137.42, p <0.01); and cohesion (β = –0.134, t _(4560)_ = –8.05, p <0.01), conflict (β = 0.143, t _(4560)_ = 9.53, p <0.01) and control (β = 0.206, t _(4560)_ = 14.44, p <0.01) were significantly associated with depressive symptoms.

**Table 4:** Hierarchical linear regression analysis of the relationship between family environment and depressive symptoms.

		β	t	p	F change	R^2^	R^2^ change
Step1					34.099	0.036	0.036
	Age	0.056	2.782	0.005			
	Gender	-0.009	-0.610	0.542			
	Ethnicity	0.000	0.006	0.995			
	Major satisfaction	0.182	12.515	<0.001			
	Grades	-0.071	-3.549	<0.001			
Step2					31.183	0.068	0.032
	Age	0.029	1.428	0.153			
	Gender	-0.019	-1.300	0.194			
	Ethnicity	0.001	0.094	0.925			
	Major satisfaction	0.167	11.583	<0.001			
	Grades	-0.054	-2.701	0.007			
	Family economic status	0.057	3.690	<0.001			
	Maternal literacy	-0.073	-3.968	<0.001			
	Paternal literacy	-0.018	-0.991	0.322			
	Family structure	-0.037	-2.339	0.019			
	Parent relationship	0.137	8.547	<0.001			
Step3					137.417	0.230	0.162
	Age	0.019	1.021	0.307			
	Gender	-0.016	-1.231	0.218			
	Ethnicity	0.006	0.459	0.646			
	Major satisfaction	0.131	9.941	<0.001			
	Grades	-0.044	-2.441	0.015			
	Family economic status	0.044	3.118	0.002			
	Maternal literacy	-0.065	-3.827	<0.001			
	Paternal literacy	-0.023	-1.369	0.171			
	Family structure	-0.031	-2.146	0.032			
	Parent relationship	0.108	7.422	<0.001			
	Cohesion	-0.134	-8.054	<0.001			
	Conflict	0.143	9.528	<0.001			
	Achievement	-0.053	-3.683	<0.001			
	Intellectual-cultural	0.054	3.749	<0.001			
	Active-recreational	-0.083	-5.861	<0.001			
	Organization	-0.077	-5.184	<0.001			
	Control	0.206	14.437	<0.001			

Note: Gender: 1 = female, 2 = male; Ethnicity: Han = 1, other ethnicity = 2; Major of satisfaction: 1 = yes, 2 = moderate, 3 = no; Grades: 1 = first, 2 = second, 3 = third, 4 = forth; Family income situation: 1 = good(good and moderate), 2 = poor; Maternal/paternal literacy: 1 = primary school, 2 = junior school, 3 = senior school,4 = college or higher; Family structure:1 = non-intact, 2 = intact; Parent relationship: 1 = good, 2 = moderate, 3 = poor; other variables were continuous variables.

## Discussion

As we hypothesized, the overall results indicated that family environmental factors were associated with depressive symptoms in Chinese university students. Specifically, the hard family environmental factors—poor parent relationship, poor family economic status, lower levels of maternal literacy, lower levels of paternal literacy and non-intact family structure—were associated with higher levels of depressive symptoms. The soft family environmental factors—conflict and control—correlated positively with depression level and lack of family cohesion was closely associated with depressive symptoms, even after controlling for other important associates of depression. Hierarchical regression analysis indicated that between family environment and depression, soft family environmental factors correlate more strongly with depression than hard family environmental factors. These findings underline the significance of family environment dimensions as risk factors of depression among university students in China.

Among the hard family environmental factors, the strongest indicator of depression was the parent relationship. We found that the worst parent relationship was associated with the higher level of depression. In line with our study, parent marital discord has been consistently regarded as a risk factor of depression in adolescents [20]. Most Chinese families have only three members including two parents and one child. In the event of conflict between parents, the child may more easily exhibit signs of behavioral distress such as sadness, fear, guilt, shame, worry and physiological reactions [34]. Emery et al. reported that marital discord has a more deleterious effect on child functioning than parental divorce or separation [35]. Consistent with their study, we also found that the parental relationship had a greater impact on depression than family structure.

Our findings showed that maternal literacy significantly influenced the depression levels of university students but paternal literacy did not influence. Low parent education with increased risk for depressive symptoms among adolescents was found in several studies [20,36,37,38]. For example, Ibrahim et al. reported that maternal education is a predictor for depression in university students [38]. In a Swedish longitudinal study, Wirback et al. found that the risk of depressive symptoms increased in adolescents whose parents had low education [37].Importantly, Sonego et al. reported that maternal education showed stronger association with depression than did paternal education when parent had a sub-university education [36].The possible explanation for increased risk in university students could be that mothers with a high level of education have good employment opportunities to supplement the family income and are more likely to contribute to a stable financial situation than those with poor education [9]. Moreover, Chinese culture traditionally emphasizes the responsibility of the husband to earn money and the wife to care for the child. In this scenario, mothers have more time to communicate with the child and have a better chance to understand his or her thoughts. Mothers with a higher level of education may be better at communicating with both husband and child, coping with stress and generally creating a harmonious psychological environment in the family. These mothers are prone to paying close attention to the psychological condition of the child and actively communicating with the child, as well as offering better emotional and practical support [39].We also found that some university students may have a higher risk of depression due to the poor economic condition of their families. Students in poor economic conditions routinely experience high levels of chronic stress because of economic problems and may therefore be more vulnerable to psychological issues including depression [3].

As we hypothesized, soft family environmental factors were a major factor in the prevalence of depressive symptoms among Chinese university students. Students experiencing high levels of family conflict and control or low family cohesion reported more depressive symptoms. Consistentwith our study, Moos reported that a supportive and cohesive family environment fosters psychological well-being [40]. In addition, individuals from families with high cohesion have been found to remain healthy even under stressful situations [23]. Cohesion and conflict mainly reflect the relationships between family members. In low cohesion and high conflict family environments, family members lack communication and do not express their opinions and emotions openly. Moreover, individuals from low cohesion families were reluctant to offer help and support and are left feeling completely alone when facing stress. Individuals who perceive less support from their family have been found to experience more depression [41]. Furthermore, according to the stress-buffering effects of positive relationships with parents, university students can ask parents for help when they encounter difficulties and experience stressful events in the context of a warm family environment; whereas students who live in the context of a hostile family environment not only receive increased pressure from the family environment, but also lack the protective factors associated with positive and effective parenting. As a result, these students have a higher risk, and indeed have a heightened incidence, of depression [42]. This finding indicated that high family cohesion and low conflict represent important factors in building a supportive family environment and preventing depression in adolescents.

We also observed a positive correlation between family control and depression, suggesting that family control can exacerbate depressive symptoms. This finding is in agreement with other studies, which found that high control is associated with depression [40]. A recent study also reported that over-control of adolescents by their parents was a risk factor for developing a psychiatric disorder later on [43]. Contradictory results were found in Nottingham University survey which reported that high levels of family control was negative associated with depression [39]. Similarly, a survey by Steptoe et al. found that high sense of personal control is protective factor for depression [20].The discrepancy results may result from the different meaning of control, family control emphasize that the family members use the fixed rules and procedures to arrange their life in our study, while in Steptoe’s and Ibrahim’s studies “control” means the ability to control life independently. Our results could be explained by the fact that the more rules and regulations to obey, the less flexibility to deal with stressful events. It may be that where families exert a strong rule control over activities that young people don’t develop a sense of being able to deal with stressful events flexibly. In an over-controlling family environment, children are deprived of the opportunities to effectively regulate their emotions and tend to suppress their expression, thoughts and emotions [44]. Suppressing emotion has been directly related to rumination, which is a vulnerability factor for depression [45].

## Study Limitations

Some limitations of this study must be acknowledged. First, the cross-sectional design makes it impossible to prove etiological hypotheses because participant factors may influence family environmental factors, such as parenting style or parental marital quality. Second, the data are based entirely on self-evaluations, which may have introduced sources of error. Third, these findings are based on Chinese university students in Harbin, therefore must be replicated and assessed in different Chinese districts.

## Conclusions

Depressive symptoms in university students in Harbin, China were closely related to family environmental factors. Soft family environmental factors—especially cohesion, conflict and control—appeared to play an important role in the occurrence of depressive symptoms. Hard family environmental factors—including family economic situation, parental relationship and maternal education—should be considered during adolescence. These findings underline the significance of the family environment as a source of risk factors for depression among university students in China and suggest that family-based interventions and improvement are crucial for reducing depression among university students.

## Supporting Information

S1 Dataset Dataset of the subsamples. (SAV)
